# Starch and tuber traits of diploid potato lines B26 and B100 and their hybrids

**DOI:** 10.1186/s12870-026-09141-1

**Published:** 2026-06-01

**Authors:** Thomas Navarro, Yordan Dolaptchiev, Oluwaseyi Bello, Ciara O’Brien, Andrés Ortiz, Jonathan D. G. Jones, David Seung

**Affiliations:** 1 John Innes Centre, Norwich Research Park, Norwich, NR4 7UH UK; 2 The Sainsbury Laboratory, University of East Anglia, Norwich, NR4 7UH UK

**Keywords:** Starch, potato, diploid, starch granule, resistant starch, source-sink, fruit, tubers

## Abstract

Diploid potato breeding enables faster genetic improvement via selection against deleterious alleles in inbred lines, unlike breeding by intercrossing tetraploid varieties. Starch is the major source of calories in potato tubers, but the starch properties of diploid lines have rarely been reported. In this study, we provide a comprehensive characterisation of tuber and starch properties in two diploid lines that are early isolates from the Solynta breeding program, B26 and B100, and their F_1_ hybrids. B100 produced fewer, but larger tubers compared to B26, and both diploid lines produced tubers that are smaller than the tetraploid variety, Clearwater Russet. The low tuber yield of B100 correlates with its high self-compatibility and fruit production. Pruning of fruits in B100 significantly increased total tuber yield per plant by stimulating more tuber initiations, but had no effect on average tuber weight, starch content or starch structure. Among the diploid, hybrid and tetraploid lines examined, there were no differences in the total starch content of tubers. Although amylopectin structure and amylose content were similar between the two diploid lines and the tetraploid comparison, B26 had elevated levels of resistant starch and a striking elongated granule morphology. Our results showcase the variation in source-sink relations and starch structure in diploid potato breeding material, demonstrating their potential for research into the genetics underpinning metabolic and quality traits.

**Supplementary Information:**

The online version contains supplementary material available at 10.1186/s12870-026-09141-1.

## Introduction

Potato (*Solanum tuberosum L.*) is the fourth most produced food crop globally, and the most produced non-cereal crop [1]. The major source of calories in potato is starch, the main carbohydrate component of the tubers. Starch is important for nutritional value as well as functional applications due to its unique physicochemical properties [2–4]. It is mainly composed by two glucose polymers, amylopectin and amylose, both made of α-1,4 linear chains and α-1,6-linked branched points. Amylopectin is the major polymer constituent, typically 67–80% of potato starch, and is highly branched. Amylose comprises the remaining 20–33%, and is primarily linear [5, 6]. Starch forms semi-crystalline, insoluble granules, which for potato have an average diameter of 40–50 μm, but ranging from 10 to 110 μm [7]. Both polymer structure and granule morphology influence the functional and nutritional properties of starch [8].

Most commercial potato cultivars are tetraploid, highly heterozygous, and self-incompatible. Their vegetative propagation through genetically identical seed tubers results in long generation cycles and pathogen contamination [9, 10]. Tetraploid potato breeding involves intercrossing varieties and screening for rare F_1_ progeny that are better than either parent, and results in low genetic gain. To tackle these challenges, significant progress has been made in developing homozygous inbred diploid lines for F_1_ hybrid seed production, accelerating genetic gain and enabling selection against deleterious alleles [11–13]. Hybrid breeding has proven successful in crops like maize and could revolutionise potato farming by enabling the use of true potato seeds, offering genetic stability, reduced storage costs, and lower disease risks compared to seed tubers [14]. Additionally, the simplified genetic background and crossability of diploid lines provide an efficient platform for studying the genetics of important traits and enable elimination of transgenes required for gene editing.

Despite their potential in potato breeding and biotechnology, there are no reports of starch traits in this novel diploid germplasm. It is therefore not known whether there is variation in key starch parameters within modern diploid breeding programs in comparison to extensively characterised tetraploid varieties. Here, we characterised the starch properties of two inbred lines from the early stages of Solynta’s diploid breeding program, B26 and B100, and two hybrids generated between these lines. Although a complete pedigree is not available, these diploid lines were derived from crosses between the diploid wild potato *Solanum chacoense* and dihaploid lines produced from tetraploid varieties [10, 15]. These lines possess useful traits for research, as B26 is readily transformed, while B100 has robust flowering that allows effective crossing. Hybrids YD2 and YD3 were originally developed as lines that combine these useful traits from their parents. We demonstrate that the two diploid lines had several distinct starch and physiological features, which can be exploited for the further study of metabolic traits and introducing them into breeding programs.

## Results

### Plant biomass and tuber yield varied among diploid lines

**Fig. 1:**
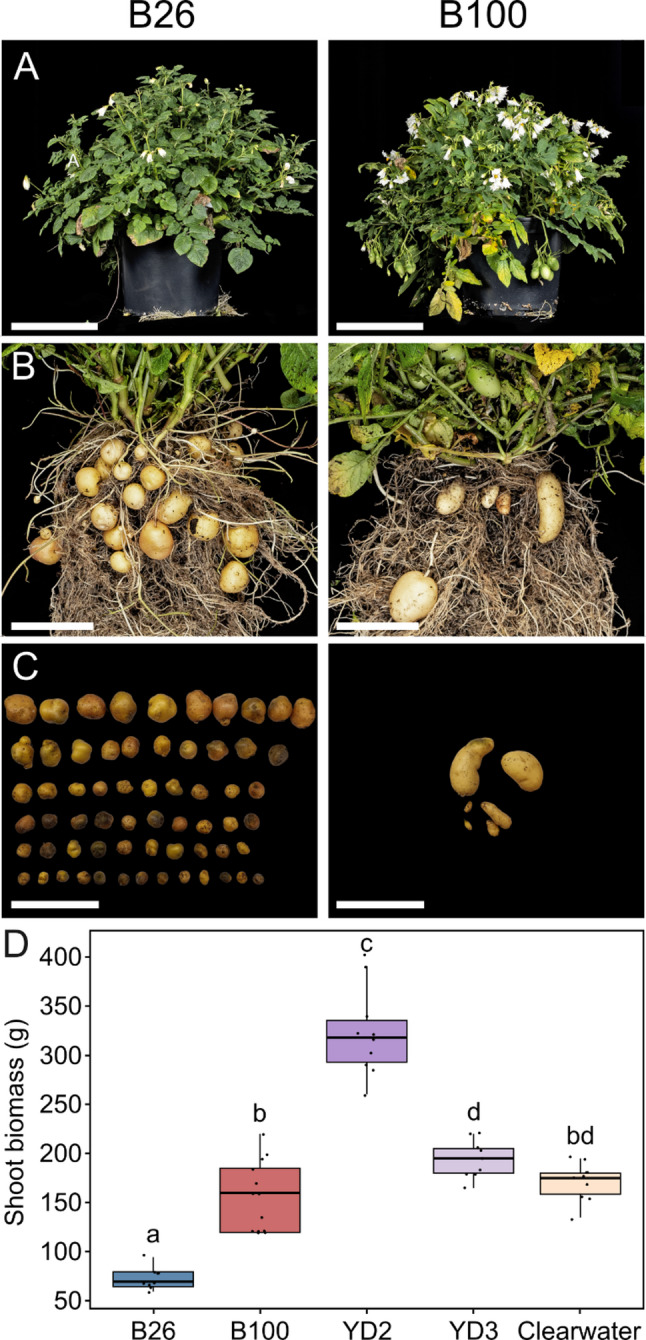
Growth and tuber phenotypes of diploid potato lines.** A** Photographs of a representative 12-week-old plants of lines B26 and B100. Bar = 15 cm. **B** Same plant as panel **A**, showing tubers after washing away the soil. Bar = 5 cm. **C** Tubers from a representative plant after harvesting (at 17 weeks). Bar = 10 cm. **D** Shoot biomass as fresh weight of total above-ground biomass measured before harvest (at 17 weeks) from diploid lines (B26, B100), diploid hybrids (YD2, YD3), and commercial tetraploid variety (Clearwater Russet). For the boxplots, the band inside the box represents the median, while the top and the bottom of the box represent the lower and upper quartiles, respectively. The whiskers represent values within 1.5× of the interquartile range. Dots represent individual data points (*n* = 9–13 per genotype), with each point representing an individual plant. Values with different letters are significantly different from each other under a one-way ANOVA with Tukey’s posthoc test (*p* < 0.05)

To assess the phenotype of the two diploid lines (B26 and B100) and their F_1_ hybrids (YD2 and YD3), plants were grown under standard glasshouse conditions. We included the commercial tetraploid, Clearwater Russet, as a previously characterised reference [16]. Under these conditions, the two parent lines differed in growth habit, with B100 plants generating many more flowers and berries than B26 (Fig. 1A). This was at least partially due to flower bud abortion in B26 (Figure S1). However, B26 had visibly more tubers than B100 (Fig. 1B). Tubers were harvested from 17-week-old plants (Fig. 1C), and shoot biomass was quantified as the total fresh weight of above-ground tissues immediately prior to tuber harvest. B100 had significantly higher shoot biomass than B26, and comparable biomass to Clearwater Russet (Fig. 1D). The shoot biomass of the two hybrids, YD2 and YD3, was significantly higher than either diploid parent.

**Fig. 2:**
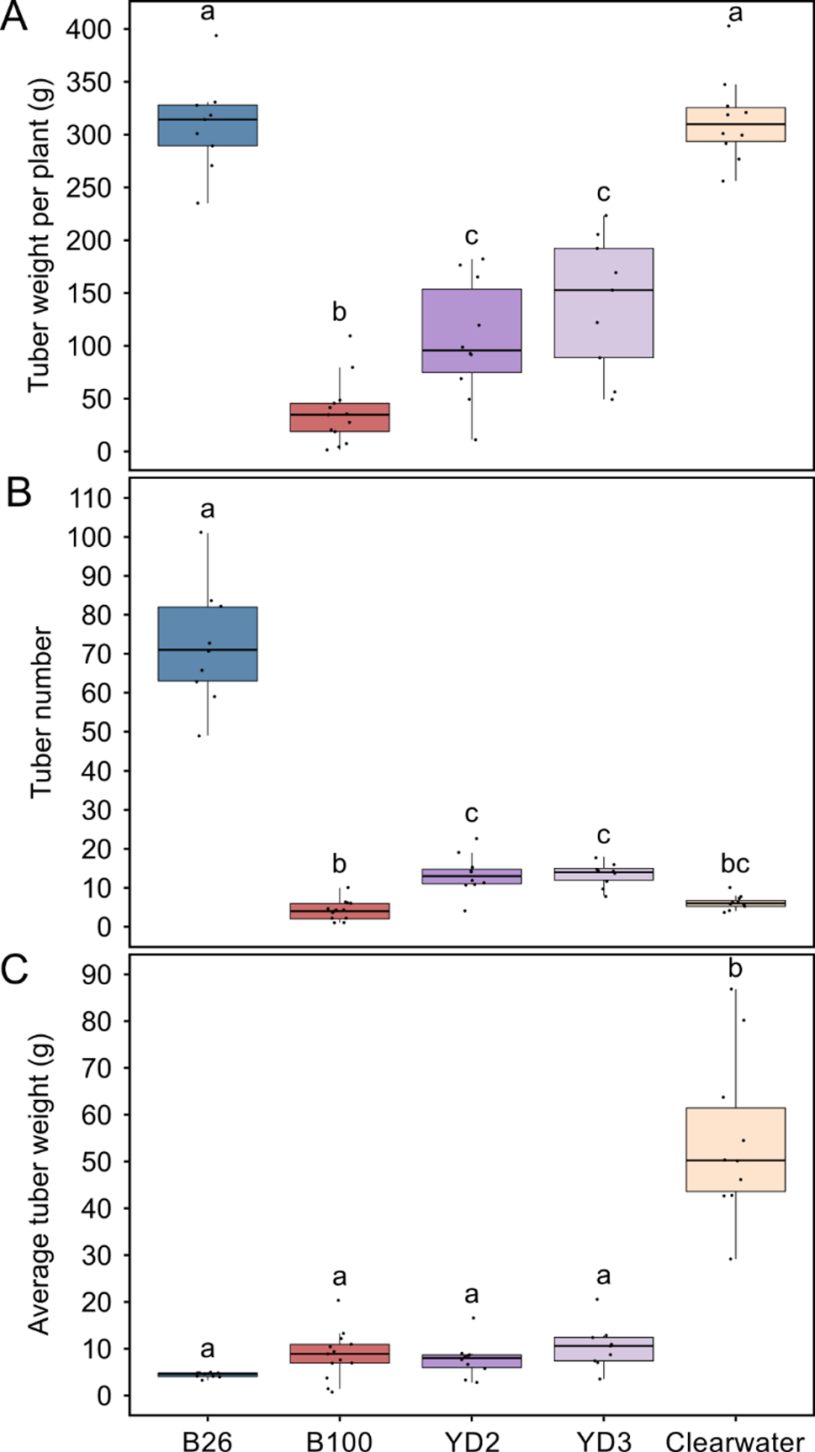
Tuber yield measurements of diploid potato lines. Tubers were harvested from 17-week-old plants of diploid lines (B26, B100), diploid hybrids (YD2, YD3), and commercial tetraploid (Clearwater Russet). **A** Total tuber weight harvested per plant. **B** Number of tubers per plant. **C** Average weight of individual tubers, calculated for each plant. For the boxplots, the band inside the box represents the median, while the top and the bottom of the box represent the lower and upper quartiles, respectively. The whiskers represent values within 1.5× of the interquartile range. Dots represent individual data points (*n* = 9–13 per genotype, with each point representing an individual plant). Values with different letters are significantly different from each other under a one-way ANOVA with Tukey’s posthoc test (*p* < 0.05)

After harvest, we quantified tuber traits, which again varied between B26 and B100 under our conditions. Overall, B100 produced significantly lower total tuber yield per plant than B26, quantified as the total weight of all tubers harvested per plant (Fig. 2A). Tuber yield per plant in B26 was comparable to Clearwater Russet. However, B26 produced extremely large numbers of tubers, with a mean of 72 tubers per plant, whereas B100 produced at most 10 tubers per plant (Fig. 2B). The mean number of tubers per plant was not significantly different between B100 and Clearwater Russet. The total tuber yield and tuber number for YD2 and YD3 were intermediate between the parent lines. Although there were no significant differences in the average weight of the diploid tubers, which was likely due to the high variability in this trait between plants. The average weight of the tubers in B26 was consistently low, between 3.2 and 5.0 g among the 9 replicate plants (Fig. 2C). This is in strong contrast to B100, where average tuber weight was more variable between replicates, from 0.7 g and up to 20.3 g. Clearwater Russet consistently produced large tubers, with an average tuber weight that was 20.5–86.8 g. B26 and B100 are therefore distinct in tuber forming habit, where B26 consistently produced many small tubers, while the tubers of B100 had a broader size range could grow larger than those of B26. However, none of the diploid lines produced tubers that were comparable in size to Clearwater Russet.

### Removing berry formation in B100 increases tuber yield

**Fig. 3:**
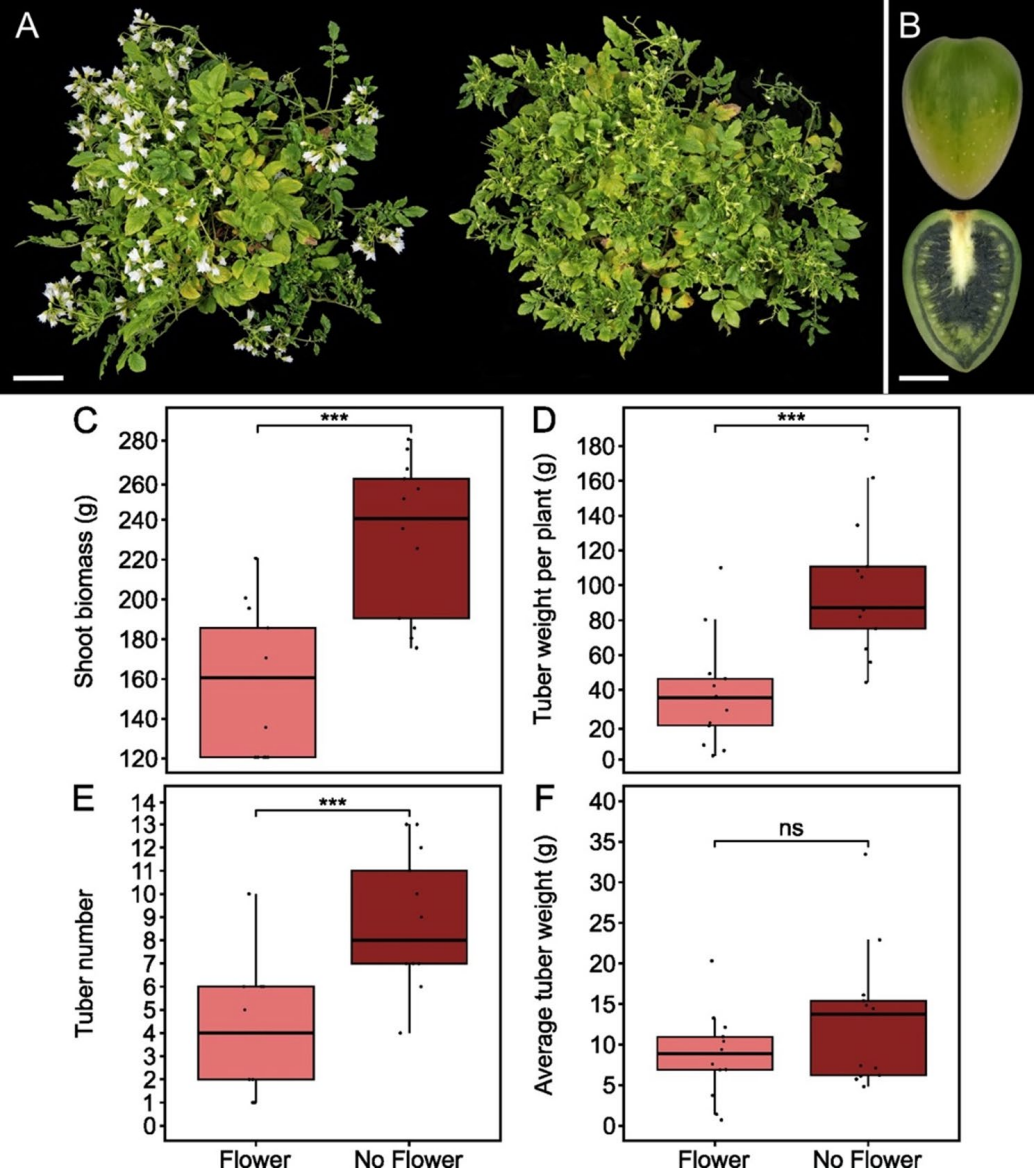
Flower removal in B100 increases shoot biomass and number of tubers.** A** Photographs of two representative 12-week-old plants from B100 without (left) and with (right) flower removal. Bar = 10 cm. **B** Photograph of a harvested berry from B100. Top half shows a whole berry, while the bottom shows a longitudinal section stained with iodine to visualise starch (stained dark). Bar = 1 cm. **C** Shoot biomass as fresh weight of total above-ground biomass measured before harvest (at 17 weeks) in plants with and without flower removal. **D** Total tuber weight harvested per plant. **E** Number of tubers per plant. **F** Average weight of individual tubers, calculated for each plant. For the boxplots, the band inside the box represents the median, while the top and the bottom of the box represent the lower and upper quartiles, respectively. The whiskers represent values within 1.5× of the interquartile range. Dots represent individual data points (*n* = 13 per genotype, with each point representing an individual plant). *** represents significance under a two-tailed t-test at *p* < 0.001. ns = not significant

In addition to the low tuber yield observed in B100, a striking feature of B100 plants was extensive flowering and fruit set, reflecting a high degree of self-compatibility in the line (Fig. 3A). The berries resembled an elongated tomato fruit (~ 3 cm long), and iodine staining in the immature berry revealed starch accumulation in the locular cavity surrounding the seeds (Fig. 3B). Berry formation was rare in the other genotypes, thus we used this unique character of B100 to test whether berries could act as a competing carbon sink during tuber development, contributing to the low tuber yield in this line. To address this, we mechanically disrupted the flowers in a subset of B100 plants to prevent berry formation and compared shoot and tuber responses to control plants where flowering and berry formation was left intact. Under our growth conditions, flower disruption led to altered plant development, increasing production of stems and leaves. This led to 43% higher shoot biomass in plants with flower disruption compared to non-disrupted plants at the time of harvest (Fig. 3C). Total tuber weight per plant was significantly higher in plants where berry formation was prevented in comparison to control plants with berries (Fig. 3D). These findings suggest that berries represent a competing sink, and disrupting their formation allows greater partitioning of carbon towards tubers and shoot biomass. Interestingly, the increased tuber yield per plant after flower disruption was primarily driven by an increase in tuber initiation. Plants with berries removed had almost double the number of tubers compared to control plants (Fig. 3E). There was no significant effect on the average tuber weight after flower disruption (Fig. 3F).

Taken together, B26 and B100 differ in growth habit under our growth conditions, in that B26 yields many tubers, while B100 yields very few. The large amount of fruit formation in B100 is one of the reasons for its low tuber yield. The diploid lines therefore offer physiological variation that is valuable for studying source-sink relations, in addition to berry and tuber initiation.

### Starch composition and structure vary among the diploid lines

**Fig. 4:**
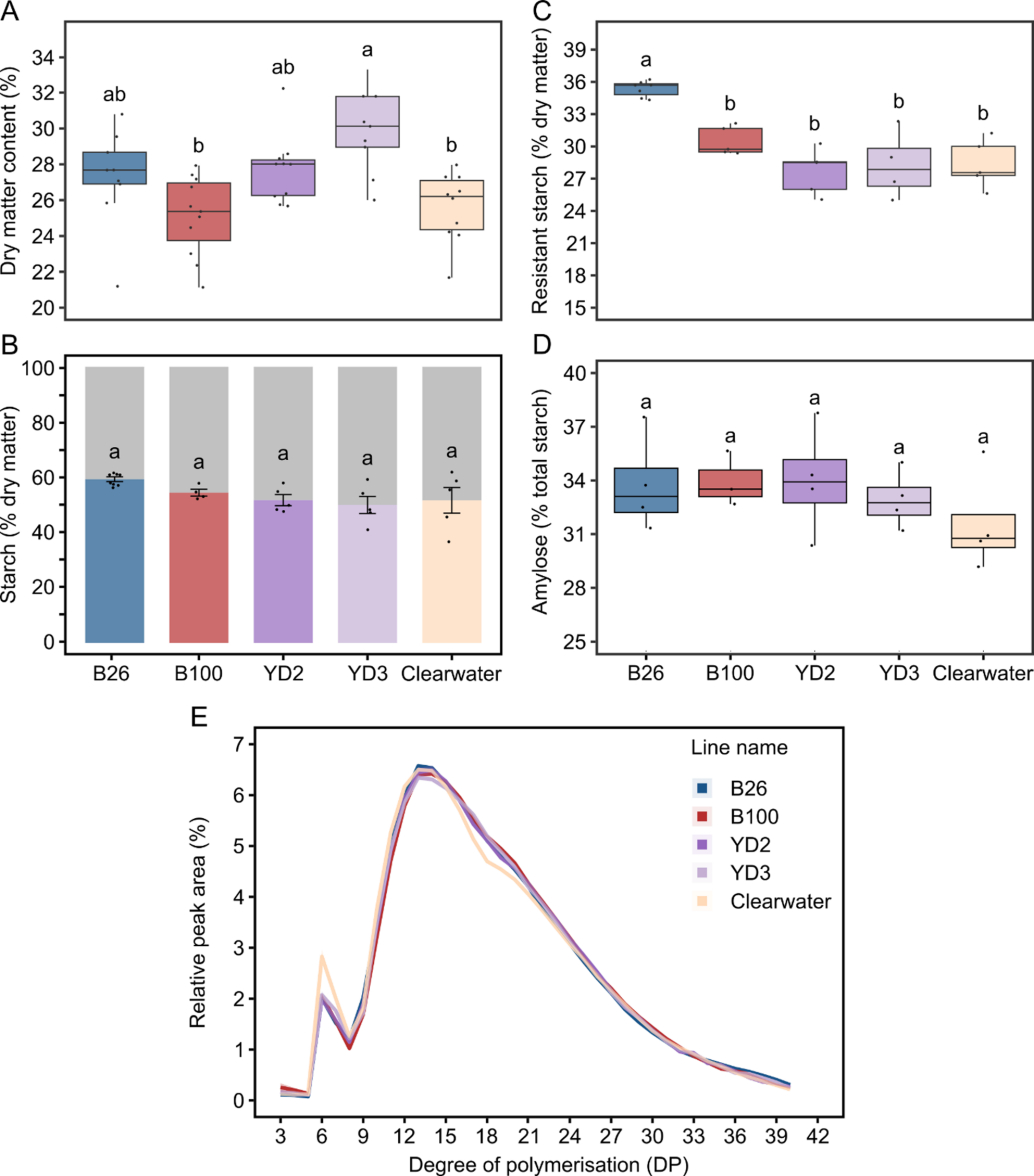
B26 has higher levels of resistant starch compared to the other diploid lines.** A** Percentage of dry matter in potato tubers in diploid lines (B26, B100), diploid hybrids (YD2, YD3), and commercial tetraploid (Clearwater Russet). Dots represent individual data points (*n* = 9–11 per genotype, with each point representing an individual plant). **B** Total starch content extracted from the dry matter fraction of potato tubers. Stacking of bars represents the different fractions of starch with the bottom chart in colours representing resistant starch, the middle dark grey chart for non-resistant starch, and the top light grey chart for non-starch fraction. **C** Boxplot of resistant starch fraction in potato dry matter from panel **B**. Dots represent individual data points (*n* = 4–5 per genotype, with each point representing tubers harvested from an individual plant). **D** Amylose content as a percentage of total starch from potato tuber (*n* = 4 per genotype, with each point representing a different plant). **E** Amylopectin chain length distribution determined using High Performance Anion Exchange Chromatography with Pulsed Amperometric Detection (HPAEC-PAD). The relative peak values for each DP, with a solid line corresponding to the mean value, and the shaded area for the standard error. For all boxplots in this figure, the band inside the box represents the median, while the top and the bottom of the box represent the lower and upper quartiles, respectively. The whiskers represent values within 1.5× of the interquartile range. Values with different letters are significantly different from each other under a one-way ANOVA with Tukey’s posthoc test (*p* < 0.05)

Since starch properties have not been characterised in diploid potato lines, we aimed to quantify major starch parameters between B26 and B100 and its hybrids. We first quantified dry matter content and total starch content in the lines. Although there were some significant differences between the lines in dry matter content, the differences were relatively small (< 5%), and there were no significant differences in total starch content. However, when we quantified the amount of resistant starch, which is an important starch parameter for tuber nutritional properties, B26 had significantly more resistant starch than the other lines (15.0% increase relative to B100, 24.7% increase relative to Clearwater Russet). As increases in resistant starch can occur due to increases in the amount of amylose, we quantified amylose content (Fig. 4D). The average amylose content ranged from 32.9% to 34.0% in the diploids and hybrids, compared to 31.6% in Clearwater Russet. These values are on the higher end of the expected amylose content for potato as reported in previous literature [17–20]. However, there were no significant differences between our diploid lines and Clearwater. Therefore, the increased levels of resistant starch in B26 are likely due to starch properties other than amylose content.

To assess for other changes in polymer composition and structure, we determined the amylopectin chain length distribution using High Performance Anion Exchange Chromatography with Pulsed Amperometric Detection (HPAEC-PAD). There were no differences in amylopectin chain length structure between the diploid lines. However, all the diploid lines had an altered chain length distribution compared to the tetraploid, with a lower proportion of short chains (DP 6 & 7) and increased proportion of medium chains (DP 16–21) (Figure S2B and S2C).

**Fig. 5:**
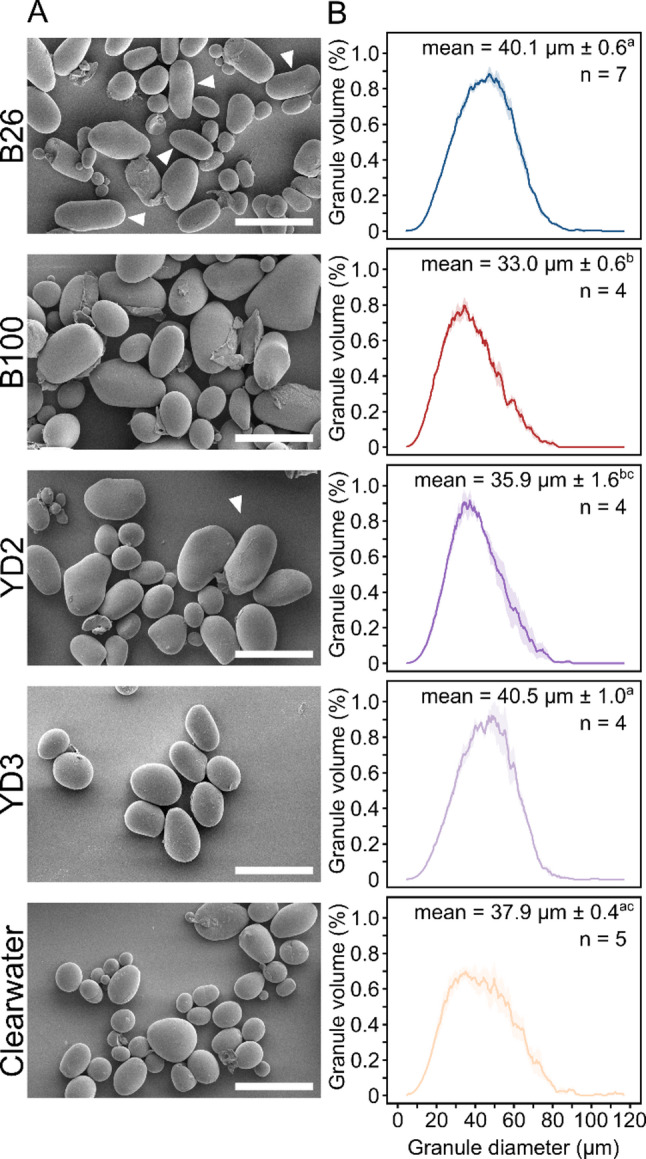
B26 starch granules are large and elongated.** A** Scanning Electron Microscopy (SEM) of purified starch granules from diploid lines (B26, B100), diploid hybrids (YD2, YD3), and commercial tetraploid (Clearwater Russet). Arrows highlight elongated starch granules in B26 and YD2. Bar = 50 mm. **B** Starch granule size distribution plots showing percentage of relative granule volume against granule diameter. The line represents the mean distribution from *n* = 4–7 plants per genotype, while shading represents the standard error. The mean granule size ± standard error for each genotype is indicated on each panel. Values with different letters are significantly different from each other under a one-way ANOVA with Tukey’s posthoc test (*p* < 0.05)

Finally, we examined the morphology of the starch granules. We first quantified starch granule size distributions by analysing purified starch granules on the Coulter counter. Potato starch granules are typically elliptic in shape [16, 21]. Using Scanning Electron Microscopy (SEM) on purified starch granules, we observed that B100 and Clearwater Russet showed this typical granule morphology of potato starch (Fig. 5A). Surprisingly, B26 starch granules showed a distinct morphology, where the granules were more elongated. To our knowledge, such a granule morphology has never been reported for storage starches.

**Fig. 6:**
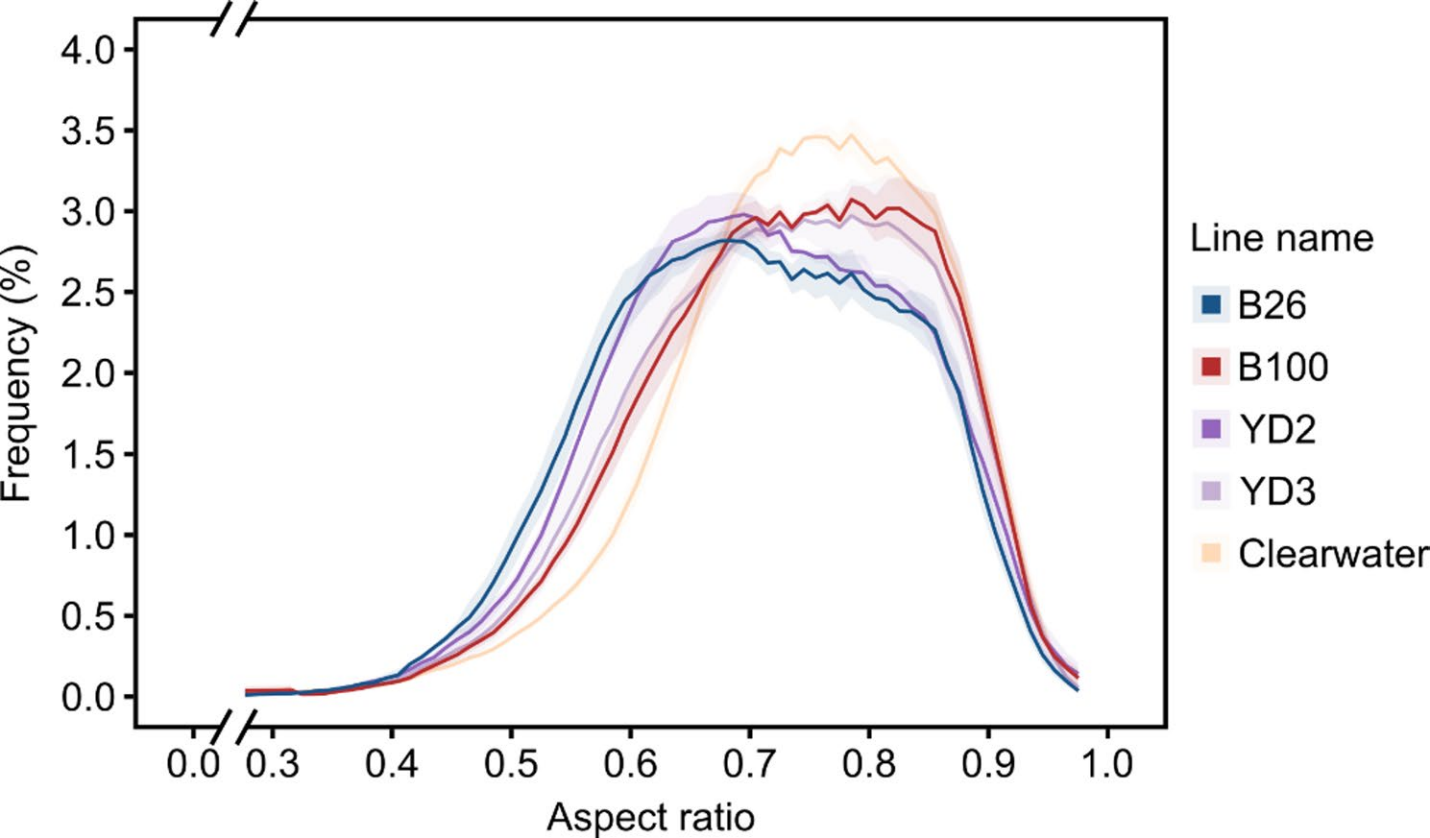
Elongated starch granule phenotype segregated in YD2 and YD3 hybrids. QICPIC particle shape analysis of diploid lines B26, B100, YD2 and YD3, and Clearwater Russet as a tetraploid comparison. The frequency of events is plotted against the aspect ratio (0 < ψ_A_ ≤ 1) with the solid line corresponding to the mean value, and the shaded area for the standard error (*n* = 3 replicate starch extractions, each from tubers harvested from separate plants)

For a quantitative assessment of starch granule morphology, we used particle size analysers. Firstly, we used the Coulter counter to quantify granule size distributions by measuring particle volume (Fig. 5B). The granules followed a unimodal distribution in all genotypes. However, B26 had significantly larger mean granule size than B100. For the hybrids, the mean granule size of YD2 resembled that of B100, while YD3 resembled that of B26. Clearwater Russet had mean granule size that was similar to B26, but significantly larger than B100. Secondly, we used the QICPIC analyser, which uses imaging to quantify granule shape parameters. The degree of elongation in starch granules can be quantified using the aspect ratio, which is the length of the minor axis divided by that of the major axis, such that the aspect ratio decreases from 1 as granules become more elongated [16]. All lines showed a broad distribution of aspect ratios. However, as we expected, the distribution of aspect ratio was similar between B100 and Clearwater Russet, but shifted towards lower aspect ratios in B26, suggesting that the granules in B26 are more elongated. Interestingly, YD2 followed the same pattern as B26, while YD3 behaved like B100 (Fig. 6). The most likely explanation is that the elongated granule trait in B26 is regulated by a single dominant, heterozygous allele that segregated in the hybrid progeny.

## Discussion

In this study, we demonstrated that two diploid potato lines, B26 and B100, have diverse physiological and tuber starch characteristics. Under our growth conditions, the two lines contrasted each other in traits such as tuber number, fruit set, resistant starch content, and starch granule morphology (Figs. 2, 4 and 5, and 6). Although these lines still have some level of heterozygosity, compared to commercial tetraploids they have less allelic variation, a smaller ploidy number, and can be crossed [10]. We show that in addition to this genetic amenability, these lines have phenotypic variation in physiological and tuber quality traits. Therefore, these lines may represent useful materials for future genetic studies of physiological and tuber quality traits.

### Diverse tuber traits in diploid potato as a resource to study tuberisation

B26 and B100 had distinct growth habit and tuber phenotypes, despite coming from the same breeding program. Tuber size in all diploid lines was much smaller than the commercial tetraploid, Clearwater Russet (Fig. 2C). However, the number of tubers in B26 was strikingly high compared to the other lines, and although each tuber was relatively small, the total tuber yield per plant (by weight) was comparable to Clearwater Russet (Fig. 2A). This suggests that tuber initiation is more active in B26 compared to the other lines. By contrast, B100 had the lowest number of tubers and total tuber yield per plant among the lines. We demonstrated that this was partially due to the high level of flowering and fruit set in this line, since removing flowers doubled the total tuber yield per plant (Fig. 3D).

Interestingly, the increase in tuber yield in B100 after flower removal was primarily due to an effect on tuber initiation. Flower removal doubled the number of tubers compared to the plants with flowers/berries intact but had no impact on the average weight of tubers. There was also no effect on starch content and composition. The effect of fruit set on tuber production is generally poorly understood, as studying it requires genotypes with substantial berry production. Most commercial potato varieties produce very few berries, and therefore fruits are unlikely to represent a substantial competing sink in those lines. A previous study generated substantial variation in fruit set by crossing tetraploid lines with Phureja, but flower removal in this background had no effect on tuber yield [22]. Other high fruit-yielding varieties, also derived from crosses with Phureja, were subjected to a similar flower removal experiment, and this increased tuber yield in some but not all varieties, and not in all environments [23]. Where total tuber yield increased, it was not determined whether the increase occurs via more tubers, larger tubers, or both. Another study found that preventing fruit formation in a heavy fruit-bearing cultivar increased tuber yield by increasing tuber size, tuber number, and dry matter content [24]. This contrasts B100, where the primary effect of fruit removal was on tuber number. However, these data were from a field experiment in Ethiopia, which represents a very different environment to our study. Thus, these contrasting findings could be due to the different environments, but also the varieties used. Since the frequency of fruit formation was not quantified in the current or previous work, it is difficult to compare the extent to which berries represent a competing sink between the different varieties tested. However, B100 is an ideal variety for study of sink-source relationships, particularly for investigating the relationship between resource allocation and tuber initiation. For example, it is known that sucrose can stimulate stolon initiation, but the underlying molecular mechanisms are unknown [25]. The induction of tubers via fruit removal in B100 may represent an in vivo system where the effect of sucrose can be explored.

It is also important to note that the number of tubers produced by B26 cannot be explained by differences in resource allocation alone, as removal of flowers in B100 did not increase tuber numbers to the level of B26. This is at least partly due to flower removal promoting more vegetative growth (Fig. 3C), which is consistent with previous work [24], and prevents all assimilates from being diverted into tuber formation. Additionally, it is likely that the observed tuber yield difference between B26 and B100 is also caused by unknown genetic variation. Both diploid lines originate from crosses with *S. chacoense*. *S. chacoense* is a wild potato from the group petota, which has wild characteristics, including enhanced vegetative growth (which acts as a competing sink), berry formation and relatively low tuber yields. B26 and B100 may have different introgressions from *S. chacoense*, which contribute to the degree to which these wild characters have been inherited. There may also be variation in the molecular mechanism of tuber initiation in B26, leading to the high number of tubers.

### Unique genetic variation in B26 leads to novel starch

Profiling starch in these diploid lines revealed novel phenotypes that could be used to improve tuber quality. Dry matter content of tubers only varied slightly among the lines, and there were no differences in total starch content (Fig. 4B). However, there were two key features of the starch structure in B26 that was different from the other lines - high resistant starch and elongated granule morphology. High resistant starch in potato can arise due to high amylose [26], yet our results show no significant differences in amylose content across diploid lines compared to Clearwater (Fig. 4D). We did notice a trend of higher amylose content in the diploid lines, which can be attributed by the variation of measurements; however, all these values fall within the upper range of the expected 20% to 33% amylose content in potatoes as reported in literature [17–20]. Therefore, we suggest that the high resistant starch content in B26 is due to other factors. Granule size also has an influence on digestibility, where larger granules digest more slowly in vitro [27]. Although B26 granules were significantly larger than those of B100, size alone cannot explain the elevated resistant starch since YD3 granules were equally as large as B26 granules, but YD3 had normal resistant starch levels (Figs. 4 and 5). The elongated shape alone cannot explain the high resistant starch either, since YD2 granules had the same shape profile as B26 granules without having elevated resistant starch (Figs. 4 and 6). The fact that the two hybrids inherited either the granule shape or size characteristic of B26 suggests that different genetic loci control these two traits. It is possible that other properties of the B26 starch granules, including smoothness and structural changes that form during digestion, affect digestion rates [28].

The genetic variation underpinning the unique granule shape of B26 is not known. The segregation of the elongated granule morphology phenotype among the hybrids YD2 and YD3 suggests that a single dominant heterozygous allele in B26 may be responsible in shaping the elongated granules. This could be further studied by generating F_2_ progeny from YD2, which will likely segregate for the phenotype. Analysing more hybrids from the B26 ✕ B100 cross with quantitative granule shape analysis, such as the QICPIC (Fig. 6), could allow mapping the genetic variation responsible for the phenotype. A possible candidate gene is PROTEIN TARGETING TO STARCH 2b (PTST2b), which was recently implicated in the control of granule shape in potato [16]. PTST2b specifically affects granule shape but not granule size, and is required for anisotropic growth of granules since reducing its expression leads to near-spherical starch granules.

### Limitations and future perspectives for diploid hybrid breeding

In this study, we demonstrated that our two diploid lines, B26 and B100, had distinct growth and tuber starch characteristics. However, it is important to note that our findings are limited to the genotypes that we have studied and our growth conditions. Plants were grown under standard glasshouse conditions, and differences in growth and tuber-bearing characteristics are likely observed in different environments (such as the field). Also, it is not possible to make general comparisons between diploid and tetraploid lines, since there is substantial genetic variation among diploid and tetraploid varieties. Further, B26 and B100 are early derivatives of the Solynta breeding program, and do not represent latest commercial diploids. However, even with our single growth condition and few selected varieties, we were able to capture variation in tuber and starch properties among the diploid lines, demonstrating the utility of this germplasm for further molecular studies and breeding.

Hybrid breeding revolutionised crop breeding during the 20th century, especially in maize [14], but the concept was perceived as unachievable in potato due to its tetraploid, heterozygous genome. The development of inbred diploid lines was facilitated by the discovery of the *Sli* (S-LOCUS INHIBITOR) from wild varieties conferring self-compatibility [29, 30], allowing breeding of F_1_ hybrid potatoes [11]. Since then, research has been dedicated to improving diploid lines for improved varieties [10, 31]. Hybrid breeding also allows use of true potato seeds (TPS) - reducing storage costs and disease risks compared to seed tubers [14]. Our study demonstrates that diploid germplasm has variation in physiological and starch phenotypes, making them excellent models for studying tuber yield and quality traits. Further, the germplasm contains useful traits, such as elevated resistant starch, that can be incorporated into breeding program. Given that the variation that was observed in just two lines, analysing more lines is likely to reveal more valuable traits for genetic studies and breeding.

## Materials and methods

### Plant growth, flower disruption and tuber harvesting

The diploid research lines, B26 and B100, were obtained from Solynta, Wageningen, Netherlands. The F_1_ hybrids of B26 and B100, named YD2 and YD3, were obtained from Yordan Dolaptchiev, The Sainsbury Laboratory, Norwich, UK. The commercial tetraploid line Clearwater Russet was obtained as a nuclear stock from Science & Advice for Scottish Agriculture (SASA), Edinburgh, UK with permission from the Potato Variety Management Institute (PVMI), Moscow, Idaho, USA. Tissue culture cuttings of the plants were rooted in MS media with 3% sucrose.

Plants were grown in glasshouses at John Innes Centre, Norwich, United Kingdom between November 2023 to April 2024, supported with 600 W LED lights with 16 h light at 20 °C and 8 h dark at 16 °C. Individual plants were grown in 5 L pots filled with compost (65% peat, 25% loam, 10% grit, 3 kg/m^3^ dolomitic limestone, 1.3 kg/m^3^ pg mix, and 3 kg/m^3^ osmocote exact). Each individual plant was considered a biological replicate in our analyses, with 9 replicates grown for genotypes B26 and YD3, 10 replicates for genotypes YD2 and Clearwater Russet, and 26 plants were grown for B100. The latter group was divided in two groups of 13 plants for the experiment with the removal of flowers. All genotypes were distributed across the glasshouse bench using randomisation with Python, but in a blocked design to ensure that B100 plants with and without flower removal remained adjacent to each other.

Plants were grown for 17 weeks, then shoot biomass was measured using a spring scale (removing berries where present before measuring), and all tubers within the pot were harvested. Flower disruption on selected B100 plants was performed by mechanically removing all parts above the calyx using tweezers: including corolla, stamens, and ovaries. The process was performed 3 times a week for each plant until harvest.

### Starch staining in berries

Berries from B100 plants were harvested from 12-week-old plants, cut longitudinally, then incubated for 5 min in a 1:5 solution of Lugol’s iodine solution to deionised water.

### Tuber yield, dry matter content and sampling

Tuber number per plant and total tuber weight per plant was determined for each plant after harvesting. The 3 largest tubers were sampled for each biological replicate, where the tubers were diced, mixed and lyophilised for 3 days. The weight of the samples was measured before and after lyophilisation to determine the dry matter content, expressed as the percentage of dry weight compared to the fresh weight. Freeze-dried tuber samples were ground to flour using a Krups KM75 coffee grinder and stored at room temperature for starch analyses.

### Starch purification of potato tubers

Starch granules were purified by incubating ~ 300 mg of the lyophilised potato flour in 5 mL 0.5 M NaCl on ice for 30 min. Suspensions were filtered through a 100 mm nylon mesh with an additional 5 mL of water, before centrifugation for 15 min at 2500*g* at 10 °C. The starch pellet was washed three times in 90% (v/v) Percoll solution (50 mM Tris-HCl, pH 8), with centrifugation for 15 min at 2500*g* at 10 °C between each wash; then washed three times in 2% (w/v) SDS with centrifugation for 5 min at 3200 g and room temperature between each wash; then washed three times in cold acetone with centrifugation for 5 min at 3200 g and 4 °C between each wash. The pellet after the final wash was air dried.

### Characterisation of starch granule morphology

For analysis of starch granules using Scanning Electron Microscopy (SEM), purified granules were dried onto a glass cover slip and mounted onto an SEM stub. Samples were sputter coated with gold (8–9 nm) using a ACE600 sputter coater (Leica, Newcastle upon Tyne, UK) and then visualised using a Nova NanoSEM 450 (FEI, Hillsboro).

For analysis of volumetric granule size distributions, purified starch granules were analysed on the Multisizer 4e Coulter counter (Beckman Coulter), fitted with a 200 μm aperture. For each sample, approximately 5 mg of starch was suspended in 500 µL of Isoton II electrolyte solution and incubated at room temperature with mixing for 30 min. The sample was further diluted in 100 mL electrolyte and relative volume vs. diameter data was collected for a minimum of 50,000 particles per sample.

For analysis of particle shape (aspect ratio) distributions, we used a dynamic image analysis sensor QICPIC with wet dispersion unit LIXELL (Sympatec) and an M5 optical module with a particle size optimum range of 16 to 1252 μm. For each sample, approximately 50 mg of purified starch was diluted in 150 mL of deionized water. Imaging was performed at 80 FPS for 30 s with an optical concentration of 0.2%. The Aspect ratio ψ_A_ (0 < ψ_A_ ≤ 1) is defined by the ratio of the minimum to the maximum feret diameter ψA = x_Feret min_ / x_Feret max_.

### Total, resistant starch and amylose content

Resistant and non-resistant starch fractions were assayed using the Resistant Starch Assay Kit (K-RSTAR; Megazyme). We followed the manufacturer’s protocol with minor changes by scaling down sample size to 10 mg of potato flour and adjusting reagent volumes accordingly. Total starch was calculated as the sum of both starch fractions. Amylose content was measured from purified potato starch as previously described [32] using potato type III amylose (Sigma, A0512) as a standard.

### Amylopectin chain length distribution

Debranched starch samples were prepared from the starch based on the method as previously described [33], but with modifications: 0.2 mg of purified starch was suspended in 450 µL of water and boiled for 15 min to gelatinise the starch. The starch was debranched with 0.02 U isoamylase from Pseudomonas (Megazyme) and 1 U pullulanase M1 from *Klebsiella planticola* (Megazyme) in a 500 µL reaction in 10 mM sodium acetate, pH 4.8, 37 °C, 2.5 h. The reaction was boiled for 10 min to stop the reaction. The debranched starch was purified over sequential columns of AmberChrome, before analysis using High-Performance Anion Exchange Chromatography with Pulsed Amperometric Detection (HPAEC-PAD) on a Dionex ICS-5000-PAD fitted with a PA-200 column (Thermo).

### Data and Statistical analyses

Data analysis for tuber and plant traits consists of tuber number, total tuber weight per plant, shoot biomass, fresh and dry matter percentage, and average tuber weight where total tuber weight of each biological replicate was divided by its corresponding tuber number. Data analysis for starch properties includes total starch content, with resistant starch and non-resistant starch fractions, and mean granule size. Line plots for Coulter counter and QICPIC show smoothed data using a rolling average of 5 data points.

Data representation is divided in 2 groups: Group 1 showing data from all 5 lines, where B100 data is of untreated WT replicates, and Group 2 comparing untreated versus treated replicates for B100 line. All statistical analyses for Group 1 were done using One-Way ANOVA, while Group 2 analyses were done using Student’s t-test. Figures and data analysis were performed in R version 4.4.1.

## Supplementary Information

Supplementary Material 1.

## Data Availability

All data generated during this study are included in this article and its supplementary information files.
