# Supplementary material for: Starch and tuber traits of diploid potato lines B26 and B100 and their hybrids
Source: BMC Plant Biol. 2026 Jun 1;26:1299. doi: 10.1186/s12870-026-09141-1 (PMC13435901; doi:10.1186/s12870-026-09141-1)
Supplement: Supplementary file 1 [file 12870_2026_9141_MOESM1_ESM.pdf]

SUPPLEMENTARY MATERIAL

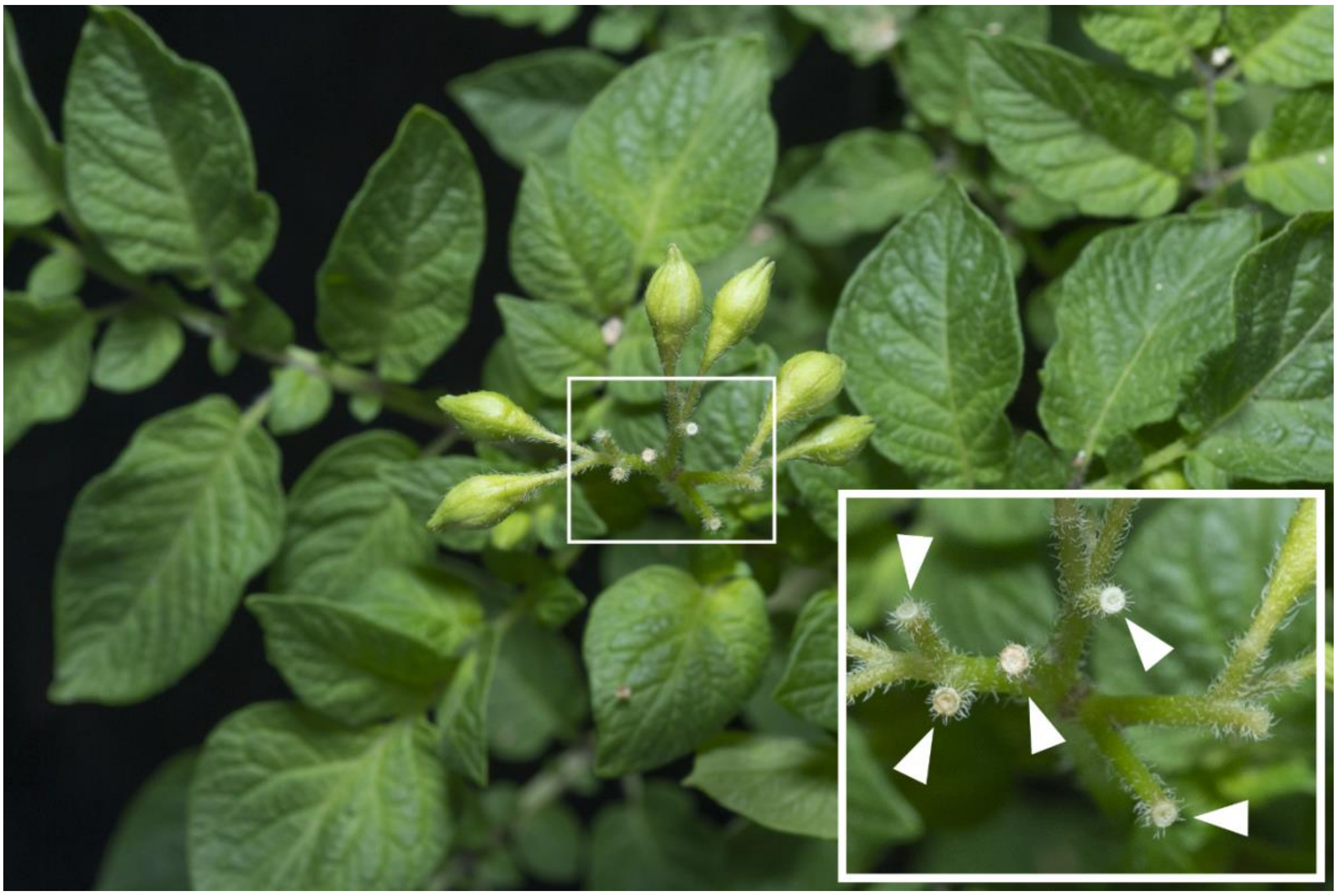

Figure S1. Flower abortion phenotype of B26. Photograph of flower buds from diploid line B26. Bud abscissions are marked with arrows.

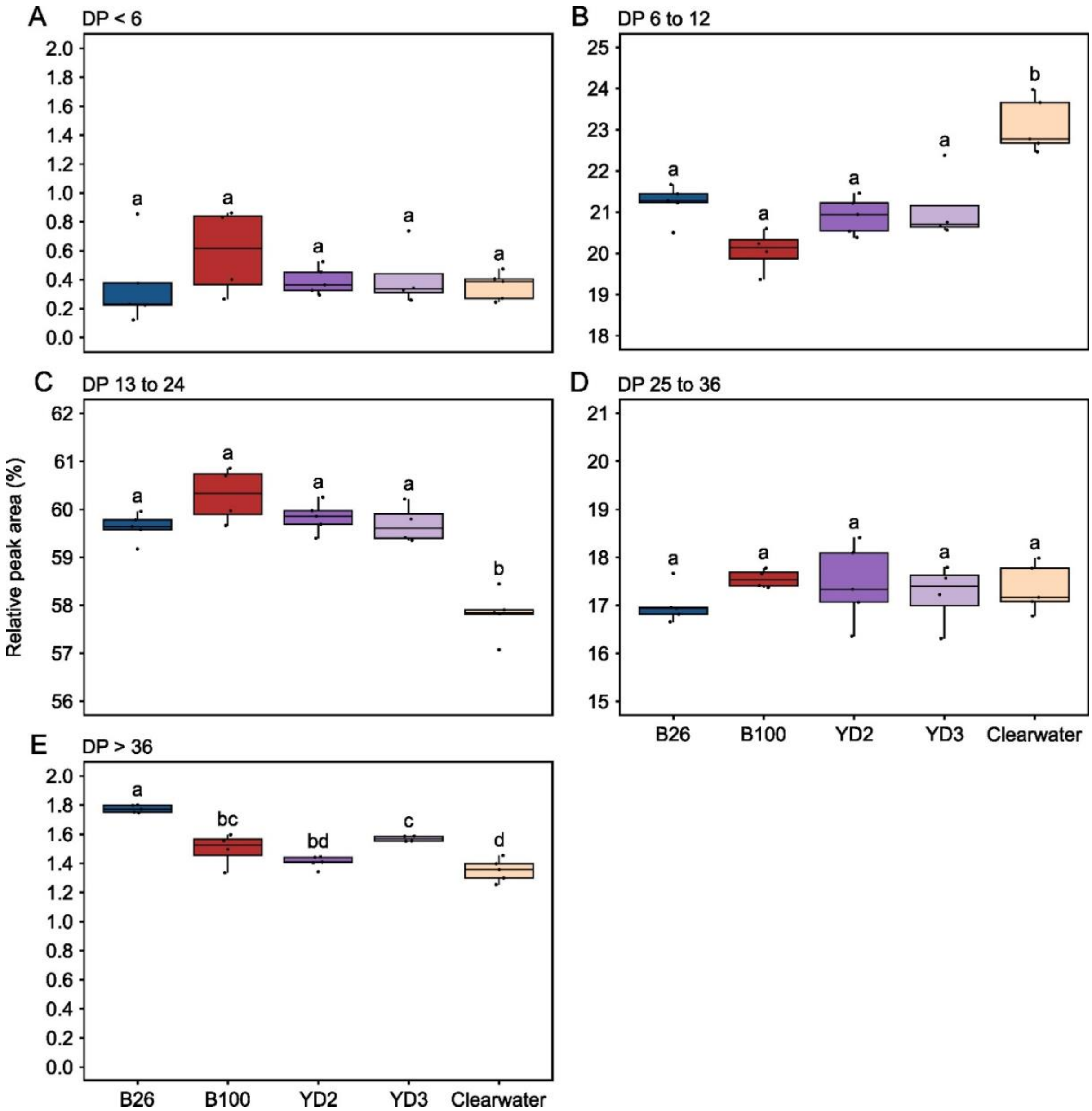

Figure S2: Diploids have an altered amylopectin chain length distribution compared to Clearwater Russet. Analysis of the amylopectin chain length distribution from Figure 4E by averaging degree of polymerisation (DP) values in five representative groups: **(A)** Chains smaller than 6 glucose units, **(B)** chains between 6 and 12 glucose units, **(C)** chains between 13 and 24 glucose units, **(D)** chains between 25 and 36 units, and **(E)** chains longer than 36 glucose units. Values represent the total relative peak area as a percentage for each group. Dots represent individual data points (n = 4-5 per genotype, with each point representing tubers harvested from an individual plant). For all boxplots in this figure, the band inside the box represents the median, while the top and the bottom of the box represent the lower and upper quartiles, respectively. The whiskers represent values within 1.5× of the interquartile range. Values with different letters are significantly different from each other under a one-way ANOVA with Tukey's posthoc test ($p < 0.05$).
